# Blake’s pouch cyst: Prenatal diagnosis and management

**DOI:** 10.4274/tjod.galenos.2020.21703

**Published:** 2021-03-12

**Authors:** Mustafa Behram, Süleyman Cemil Oğlak, Fatma Ölmez, Zeynep Gedik Özköse, Sema Süzen Çaypınar, Yusuf Başkıran, Salim Sezer, Kadriye Erdoğan, Mehmet Aytaç Yüksel, İsmail Özdemir

**Affiliations:** 1 University of Health Sciences Turkey, Kanuni Sultan Süleyman Training and Research Hospital, Clinic of Perinatology, İstanbul, Turkey; 2 University of Health Sciences Turkey, Gazi Yaşargil Training and Research Hospital, Clinic of Obstetrics and Gynecology, Diyarbakır, Turkey; 3 University of Health Sciences Turkey, Kanuni Sultan Süleyman Training and Research Hospital, Clinic of Obstetrics and Gynecology, İstanbul, Turkey; 4 University of Health Sciences Turkey, Etlik Zübeyde Hanım Women’s Health Training and Research Hospital, Clinicof Obstetrics and Gynecology, Ankara, Turkey

**Keywords:** Blake’s pouch cyst, posterior fossa, cerebellar vermis, prenatal diagnosis

## Abstract

**Objective:**

This study aimed to present the characteristic features of 19 patients who were diagnosed as having Blake’s pouch cyst (BPC) at our center.

**Materials and Methods:**

Nineteen patients diagnosed as BPC between 2015 and 2019 were included in this retrospective study. Follow-up examinations were performed using ultrasonography (US) every three weeks up to 35 weeks of gestation. Prenatal magnetic resonance imaging (MRI) was performed at the time of diagnosis or during follow-up in 13 patients. MRI or transfontanellar US was performed to confirm the diagnosis of BPC after delivery. Karyotype results of eight patients were recorded.

**Results:**

Isolated BPC was observed in 9 (47%) patients, and associated anomalies were detected in 10 (53%) patients, including seven (36%) with the central nervous system and four (21%) with cardiac anomalies. Two fetuses had abnormal karyotype analysis as trisomy 21 and 13. The MRI report of eight patients was “differential diagnosis required for Dandy-Walker complex” and only in five (26%) patients, it was reported to be compatible with BPC. Spontaneous resolution was seen in four patients. Postnatal MRI was performed in five patients, and transfontanellar US in two patients, and all MRI and US results were consistent with BPC. During the neonatal period, abnormal neurologic development was observed in four (21%) patients, and one (5%) died.

**Conclusion:**

Although the prognosis of isolated BPC is very good with healthy neurologic development until advanced ages, death in the early neonatal period and abnormal neurologic development may be observed depending on the condition of the associated anomalies.

**PRECIS:** Isolated Blake’s Pouch cyst has an excellent prognosis, with a high possibility of intrauterine resolution and healthy intellectual development.

## Introduction

Blake’s pouch is a rudimental embryologic structure of the fourth ventricular tela choroidea and it perforates in the 9^th^ or 10^th^ weeks of embryogenesis. Perforation ordinarily occurs in the foramen of Magendie. If perforation of Blake’s pouch does not occur in the foramina during embryogenesis, it leads to a ballooning of the superior medullary velum into the cisterna magna resulting in Blake’s pouch cyst (BPC) formation. During embryologic development, foramina of Luschka, having a smaller diameter than the foramen of Magendie, open later than this foramen^(1,2)^. As the perforation in the foramina of Luschka does not occur during the formation process of BPC, the fourth ventricle continues to expand with supratentorial structures until the foramina of Luschka opens and provides cerebrospinal fluid (CSF) flow from the ventricles to the cisterna magna. BPC may sometimes disappear in the third trimester due to late fenestration at the 24^th^ to 26^th^ weeks of gestation^(3)^.

Cystic malformations of the posterior fossa are frequently revealed with neuroimaging studies. An abnormal amount of CSF in the posterior fossa is classified within the spectrum called Dandy-Walker complex (DWC) or as arachnoid cysts^(1,4)^. Tortori-Donati et al.^(5)^ claimed that BPC was a different entity from cysts in DWC or arachnoid cysts. mega cisterna magna and arachnoid cysts are usually incidental findings, whereas cysts in DWC are associated with cerebellar hemisphere and other developmental anomalies related to vermis, and most commonly found together with hydrocephalus^(1,6)^. BPC has been less recognized in the radiologic spectrum among the posterior fossa’s cystic malformations because it was considered as a separate entity. However, BPC interestingly presents a broad spectrum of symptoms between showing all signs of hydrocephalus and being asymptomatic^(7)^.

This study aimed to present the associated anomalies, karyotype analysis, ultrasonographic (US) and magnetic resonance imaging (MRI) findings of 19 patients who are diagnosed as having BPC at our center over the last five years and to review the literature about BPC.

## Materials and Methods

This study was conducted retrospectively on patients admitted to the Kanuni Sultan Suleyman Research and Training Hospital Perinatology Clinic between 2015 and 2019. Nineteen patients who were diagnosed as having BPC after suspicion of posterior fossa anomalies were included in the study. The US criteria used to diagnose BPC were used as recommended by Paladini et al.^(3)^: (1) normal anatomy and normal size of vermis, (2) slight to medium rotation counterclockwise of vermis, (3) normal size of cisterna magna, (4) evidence of the wall of the BPC in the cisterna magna; the first three criteria were considered necessary for the diagnosis, and the 4^th^ criterion is supportive for diagnosis. Multiplanar 3-dimensional US (GE Voluson E6 Wide Band Convex Transducer) was used to examine the BPC and its neighboring vermis and posterior fossa. The vermian size was measured using the nomograms recommended by Viñals et al.^(8)^. Fetuses suspected of BPC before 20 weeks of gestation were re-evaluated after 20 weeks of gestation. Follow-up examinations were performed every three weeks up to 35 weeks. Prenatal MRI exams were performed at the time of diagnosis or during the follow-up period in 13 patients. All MRI examinations were conducted at a single center. In some patients where no termination was performed, MRI or transfontanel US was performed to confirm the diagnosis of BPC after delivery. US results obtained at diagnosis and associated anomalies encountered during follow-up were recorded. Karyotype results of eight patients were obtained and recorded. Delivery mode and week, postnatal neurologic development results of the fetuses were recorded. The neurologic examination included the head shape assessment, the head circumference measurement, and the cranial nerve evaluation. The upper and lower limbs (deep tendon reflexes, pathologic reflexes, movement, strength, muscular tension), abdominal reflexes, meningeal signs, superficial and deep feeling, and involuntary movements were also evaluated^(9)^.

This study was conducted after the Kanuni Sultan Suleyman Research and Training Hospital Clinical Research Ethics Committee’s gave approval and written informed consent was obtained from all participants.

### Statistical Analysis

We used the IBM SPSS 21.0 for Windows (SPSS Inc., Chicago, IL, USA) statistical package for statistical evaluation of our research data. A descriptive analysis of the records was performed following completion of the audit. Continuous variables are presented as median. Categorical variables are presented as frequencies and percentage.

## Results

This study consisted of 19 patients who were diagnosed as having BPC through prenatal US examinations after referral to our clinic due to suspicion of posterior fossa anomaly. The median gestational age at diagnosis was 23 weeks, with only two patients referred in the third trimester. Isolated BPC was observed in nine of 19 patients (47%), and associated anomalies were detected in 10 (53%) patients. Seven (36%) of the patients with multiple anomalies had central nervous system (CNS) anomalies, and 4 (21%) had cardiac anomalies. The detailed associated anomalies and other follow-up results are summarized in Table 1.

MRI was performed in 13 (68%) of 19 patients diagnosed as having BPC using US. The MRI report of eight (42%) patients was “differential diagnosis required for DWC,” and only in five (26%) patients, it was reported to be compatible with BPC.

When the spontaneous resolution of BPC was examined between the 24^th^ and 26^th^ weeks in the US follow-up, a spontaneous resolution was seen in four of 16 patients (21%). However, it could not be evaluated in three (15%) patients due to the termination of pregnancy was performed before these weeks. It was recorded that six (75%) of eight patients had a normal karyotype, and one was trisomy 13, and another patient was trisomy 21 (Down syndrome). To confirm the prenatal diagnosis of BPC in 14 patients, MRI was performed in five patients, and transfontanel US in two patients and all MRI and US results were consistent with BPC. However, the MRI or US results of other patients could not be obtained.

Termination of pregnancy was performed in five (26%) patients with multiple anomalies, 14 (74%) patients reached term. The delivery of five (35%) patients was normal spontaneous vaginal delivery at 40 weeks, and the delivery of nine (65%) patients was cesarean section (C/S) at 39 weeks. In the postnatal follow-up of the patients, no information was obtained about the neurologic development of one patient, healthy neurologic development was observed in nine (47%) patients, and abnormal neurologic development was observed in four (21%) patients. One (5%) patient died during the neonatal period due to the multiple anomalies. If the patients with healthy neurologic development after birth were examined, all patients had isolated BPC.

## Discussion

Studies on BPC have focused on the non-perforation of BPC in the foramen of Magendie. According to this theory, when the perforation of BPC in the foramen of Magendie does not occur, the cerebellar hemisphere and vermis are compressed due to increased CSF. Still, this increased pressure does not occur in the development of BPC. Therefore, most authors agree that DWC originates from a defect in the anterior membranous region, and BPC and mega cisterna magna originate from a defect in the posterior membranous region^(1,10)^.

Modern MRI methods provide essential information in identifying concomitant malformations in the differential diagnosis of BPC^(11)^. Typical radiologic findings of BPC are infra or retrocerebellar localization of the cyst, a well-developed and non-rotated cerebellar vermis, cystic dilatation of the fourth ventricle, compression of the cerebellar hemispheres to some extent, and continuity of the choroid plexus on the cyst wall^(1,11,12)^. In our study, only 5 (39%) of the 19 patients who underwent MRI in their follow-up were reported to be compatible with BPC. The reason it was reported in this way may be because radiologists have not received adequate training in the differential diagnosis of BPC. It is still challenging to discriminate mild hypoplasia from slight deformation of the cerebellar vermis in fetal and postnatal MRI^(13)^. We re-examined the MRI images and found that the images were compatible with BPC. Postnatal MRI or transfontanellar US confirmed the diagnosis of BPC in all seven patients who underwent MRI and USG after delivery.

It is seen that most articles written on BPC in the literature investigated other developmental anomalies, and most focused on embryogenesis rather than clinical results. However, the clinical presentation of BPC is extensive; it can be detected incidentally in adulthood and fatal complications can occur in the neonatal period. Cornips et al.^(1)^ presented a case series of six patients with BPC. In the case series, both a case of BPC detected incidentally in MRI screening in adulthood in a 51-year-old and a patient who died of high-pressure hydrocephalus and cholestatic anomalies at the age of one month were present. It was detected that hydrocephalus slowly developed in two patients. However, neurologic development was normal and treated with endoscopic third ventriculostomy, and there was a patient who had listeria meningitis due to compensated hydrocephalus and had healthy neurologic development. In a case report of Calabrò et al.^(12)^, two patients with BPC showed healthy neurologic development until the age of 61 and 62. They were diagnosed as having BPC when syncope attacks developed in the first patient, and headache and vertigo were seen in the second. Bontognali et al.^(14)^ presented a patient with BPC who started to show signs of cerebellar dysfunction in the 18^th^ month despite having healthy neurologic development. In the case report of Iuculano et al.^(15)^, it was reported that a patient diagnosed with prenatal BPC had no associated anomaly and showed healthy neurologic development after delivery. In our results, normal neurologic development was observed in nine (47%) of the 13 patients that reached term, which was isolated BPC; four (21%) had abnormal neurologic development and one (5%) died in the neonatal period. One of four fetuses with abnormal neurologic development had polydactyly and a dysmorphic face. One had hydrocephalus, polydactyly, and interhemispheric cyst. One had hydrocephalus and vermian hypoplasia, and the last had dysgenesis of the corpus callosum. It can be seen that fetuses with abnormal postnatal neurologic development in the postnatal period are more likely to have CNS anomalies.

In a case series of 19 patients diagnosed as having BPC using prenatal USG, Paladini et al.^(3)^ found major anomalies in eight of 19 patients (42%), and five (26%) were associated with congenital heart disease. In 12 of 19 patients, karyotype analysis results were normal, but only two were abnormal (trisomy 21). A termination was performed in eight patients (42%) and neonatal death was seen in two (10%). Eight (48%) patients reached term, and all had healthy neurologic development. The results of Paladini et al.^(3)^ were in parallel with our results. Among our fetuses with associated anomalies, seven (36%) had CNS anomalies four (21%) had cardiac anomalies, and termination of pregnancy was performed in five (26%) due to multiple anomalies. According to a case report and a meta-analysis of case series about posterior fossa anomalies, D’antonio et al.^(16)^ detected BPC in 86 fetuses from nine studies. Among these patients, the rates of associated anomalies of CNS and other than the CNS were found as 11.5% and 23.5%. Trisomy 21 was detected in only one of the 45 patients who underwent karyotype analysis. In our results, trisomy 21 and 13 were detected in two patients with BPC, and associated anomalies were observed in 10 (53%) patients. Seven (36%) of associated anomalies were CNS anomalies, and four (21%) were cardiac anomalies. In the second part of the same meta-analysis on 46 patients with BPC regarding neurologic development outcomes, no significant relationship was found between BPC and the abnormal neurologic development results^(17)^. In a study examined 105 fetuses with posterior fossa anomalies by Gandolfi-Colleoni et al.^(18)^, 32 fetuses were diagnosed as having BPC using prenatal USG, associated anomalies were detected in eight of these 32 patients, fluid accumulation in the posterior fossa with neurologic development disorder in one of 20 patients who reached term, and in one patient, abnormal neurologic development related to other anomalies were observed. Healthy neurologic development was observed in 90% of patients in 1 to 5 years of follow-up. In our results, similar to these studies, patients with BPC were most frequently associated with CNS and cardiovascular system anomalies, and all patients with isolated BPC had healthy neurologic development in post-natal life.

In the study conducted by Gandolfi-Colleoni et al.^(18)^, spontaneous resolution of BPC was observed in about one-third of 32 fetuses. In the study of Paladini et al.^(3)^, BPC regressed in 6 (55%) of the 11 patients that reached term and the vermis returned to the normal position between 24 and 26 weeks. In a case report reported by Ramaswamy et al.^(2)^, it was seen that the patients diagnosed as BPC in prenatal USG at 25 weeks had completely regressed on MRI after delivery. In our study, as in these studies, three (15%) patients could not be evaluated because termination of pregnancy was performed before 24^th^ and 26^th^ weeks, but it was observed that spontaneous resolution of BPC occurred in four (21%) of the remaining 16 patients and it continued in 12 (63%) patients.

## Conclusion

BPC, whose embryology, clinical findings, imaging characteristics, and outcomes are not known sufficiently by medical professionals, is one of the posterior fossa cystic lesions. Therefore, it may be misdiagnosed by radiologists and physicians in patients with BPC. Prognosis is excellent in patients with isolated PBC, and healthy neurologic development may be observed without any evidence until advanced adult ages. However, depending on the condition of the associated anomalies, complications such as termination, death in the early neonatal period, and abnormal neurologic development may be observed.

## Figures and Tables

**Table 1:**
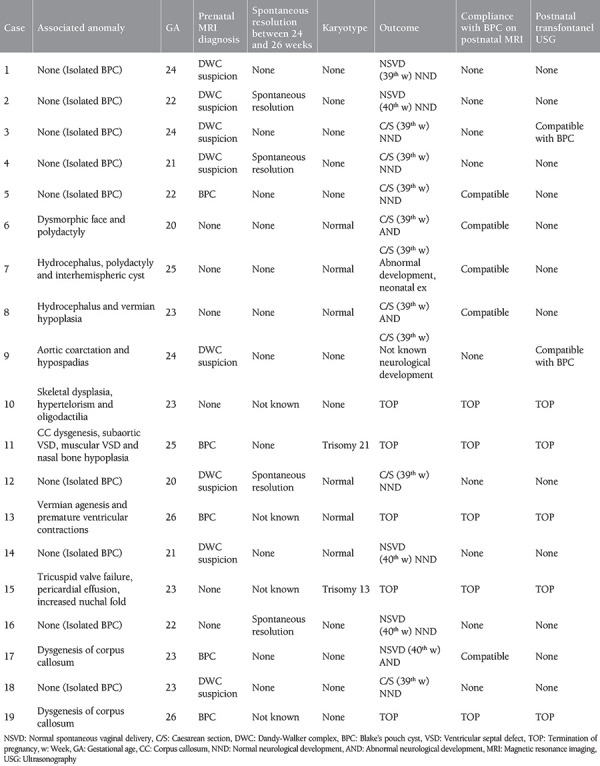
USG, MRI and follow-up results and associated anomalies of 19 patients diagnosed with BPC
